# The Opposing Role of Propionate in Modulating *Listeria monocytogenes* Intracellular Infections

**DOI:** 10.3389/fmicb.2021.721801

**Published:** 2021-09-01

**Authors:** Laura Hobbs, Leah Allen, Megan Bias, Stephanie Johnson, Hannah DeRespiris, Chantal Diallo, Loan Bui, Yvonne Sun

**Affiliations:** Department of Biology, University of Dayton, Dayton, OH, United States

**Keywords:** *Listeria monocytogenes*, propionate, macrophage, nitric oxide, anaerobic growth, macrophage morphology

## Abstract

*Listeria monocytogenes* is a Gram-positive, intracellular pathogen responsible for the highly fatal foodborne illness listeriosis. Establishing intracellular infections requires the coordinated expressions of a variety of virulence factors, such as the pore-forming toxin listeriolysin O (LLO), in response to various intra- and extracellular signals. For example, we previously reported that *L. monocytogenes* differentially modulated LLO production in response to exogenous propionate, a short chain fatty acid either used in salt form as a human food ingredient or produced endogenously by gut microbial fermentation. Therefore, propionate is likely a continuously present signal throughout the *L. monocytogenes* transmission and infection process. However, little is known about the role of propionate in modulating *L. monocytogenes-*host interactions. Here we investigated the impact of propionate treatment on *L. monocytogenes* intracellular infections using cell culture infection models. Propionate treatment was performed separately on *L. monocytogenes* or host cells before or during infections to better distinguish pathogen-versus-host responses to propionate. Intracellular CFU in RAW264.7 macrophages and plaque diameters in L-fibroblasts were measured as proxy for intracellular infection outcomes. Nitrite levels and cellular morphology were also measured to assess host responses to propionate. We found that propionate pretreatment of anaerobic, but not aerobic, *L. monocytogenes* significantly enhanced subsequent intracellular infections in both cell types and nitrite production by infected macrophages. Propionate treatment of uninfected macrophages significantly altered cell morphology, seen by longer cells and greater migration, and reduced nitrite concentration in activated macrophages. Treatment of macrophages with propionate prior to or during infections significantly inhibited intracellular growth of *L. monocytogenes*, including those pre-treated with propionate. These results showcased an opposing effect of propionate on *L. monocytogenes* intracellular infections and strongly support propionate as an important signaling molecule for both the pathogen and the host cell that can potentially alter the outcome of *L. monocytogenes*-host interactions.

## Introduction

*Listeria monocytogenes* is a Gram-positive, intracellular pathogen responsible for the foodborne illness listeriosis. While the overall prevalence is low, listeriosis is often associated with a high mortality rate of 20–30%, which results in listeriosis accounting for 19% of all deaths caused by foodborne illnesses (Scallan et al., 2011; de Noordhout et al., 2014). For high-risk individuals, such as neonates, the elderly, immunocompromised individuals, or pregnant women, listeriosis can result in sepsis, meningitis, fetal infection, and abortion (Radoshevich and Cossart, 2018). Because of the disease severity, there is a zero-tolerance policy for *L. monocytogenes* surveillance in food processing facilities or products to minimize exposure and protect high-risk populations (United States Department of Agriculture (USDA), 2014).

*Listeria monocytogenes* is difficult to control as it can grow in conditions typically used to restrict microbial growth. For example, *L. monocytogenes* can grow and survive at low temperatures (Tasara and Stephan, 2006; Chan and Wiedmann, 2008; Arguedas-Villa et al., 2010; Redfern and Verran, 2017; Bevilacqua et al., 2018) and exhibit tolerance toward freeze-thaw cycles (Azizoglu et al., 2009). It is also resistant to low pH and high salt conditions (O’Driscoll et al., 1996; Phan-Thanh and Mahouin, 1999; Duché et al., 2002; Gardan et al., 2003). Furthermore, *L. monocytogenes* is capable of forming biofilms and persister cells, allowing the pathogen to persist in food processing plants for years or even decades (Beresford et al., 2001; Møretrø and Langsrud, 2004; Buchanan et al., 2017; Jordan et al., 2018). Despite improvements in food safety over the years, multiple *L. monocytogenes* outbreaks continue to take place every year in the United States [Center for Disease Control and Prevention (CDC), 2020], making additional infection control and prevention strategies a clear priority.

As an intracellular pathogen, *L. monocytogenes* relies on coordinated expressions of a variety of virulence factors to survive and propagate inside host cells. Upon entry or uptake into a host cell, *L. monocytogenes* escapes the entry vacuole or phagosome through the activity of listeriolysin O (LLO) and phospholipases to avoid degradation (Nguyen et al., 2019). LLO, a pore-forming toxin, additionally aids in intracellular growth by altering mitochondria morphology and function (Stavru et al., 2011; Carvalho et al., 2020; Li et al., 2021). In the host cytoplasm, *L. monocytogenes* expresses surface ActA proteins and polymerizes host actin to form actin tails that allow intracellular bacteria to move through the cytosol and invade neighboring cells (Lambrechts et al., 2008). This cell-to-cell spread also involves LLO for *L. monocytogenes* to escape the double-membraned vacuoles and establish secondary infections (Gedde et al., 2000). Therefore, factors that can influence LLO production can potentially affect the overall *L. monocytogenes* pathogenesis.

Considering the variety of environmental factors *L. monocytogenes* might be exposed to, we have been focusing on the role of propionate, particularly under anaerobic conditions, in *L. monocytogenes* pathogenesis. In the salt form, sodium propionate is a Generally Recognized As Safe human food ingredient for antimicrobial and flavoring purposes (United States Food & Drug Administration (USFDA), 2020). In 2016, European Food Safety Authority provided a scientific opinion in which no safety concerns were identified for sodium propionate at concentrations up to 5,000 mg/kg in meat products (European Food Safety Authority (EFSA), 2016). Moreover, propionate is one of the main short chain fatty acids (SCFAs) produced through the gut microbial fermentation. In mammals, SCFA concentrations were reported to range from 10 to 100 mM in the colon and 0.1 to 10 mM in the blood stream (Natarajan and Pluznick, 2014; Koh et al., 2016). Moreover, as *L. monocytogenes* transitions from food matrices, the intestinal lumen, and host cell cytosol, it experiences and has to adapt to different oxygen levels. Therefore, *L. monocytogenes* is likely to be exposed to propionate before and during infections under conditions with fluctuating oxygen concentrations. How oxygen availability influences the effects of propionate during *L. monocytogenes*-host interactions remains unknown.

We previously reported that propionate supplementation significantly reduced aerobic LLO production but enhanced anaerobic LLO production (Rinehart et al., 2018), an observation that suggests propionate as a potential modulator both before and during *L. monocytogenes* intracellular infections. Therefore, in this study, we investigated and showed the impact of propionate treatment, provided to *L. monocytogenes* or host cells prior to or during infections, on the outcome of infections, thereby establishing propionate as a potential determinant in *L. monocytogenes* pathogenesis.

## Materials and Methods

### Cell Cultures

RAW264.7 macrophages (ATCC TIB-71) and murine fibroblast L-cells (ATCC CRL-2648) were cultured in DMEM (Corning 10-013-CV) supplemented with 10% (v/v) FBS (HyClone SH3091003), 50 units/mL penicillin, and 50 μg/mL streptomycin (Gibco 15070-63). Penicillin and streptomycin were removed prior to infection assays. Cells were maintained at 37°C with 5% CO_2_ in the atmosphere.

### Bacterial Cultures

*Listeria monocytogenes* strain 10403s was streaked onto fresh BHI (BD BBL 211059) plates on a weekly basis. BHI medium was prepared by filter sterilization to ensure consistency between batches. Overnight BHI liquid cultures were incubated at 37°C either aerobically with shaking at 250 rpm or anaerobically in an anaerobic chamber (Type A, Coy Laboratory) with a nitrogenous atmosphere and 2.5% of hydrogen. Overnight is defined as 16–21 h. Sodium propionate stock solutions (1 M) were filter-sterilized, aliquoted, and frozen to be thawed prior to use. To establish short exposure, untreated overnight cultures were back-diluted (1:10 for aerobic and 1:4 for anaerobic cultures) into fresh media with or without 25 mM propionate and incubated aerobically or anaerobically for 2 h. At all propionate concentrations tested, no growth inhibition on *L. monocytogenes* was observed (Rinehart et al., 2018). Culture media for back dilution under anaerobic conditions was equilibrated overnight inside the anaerobic chamber to avoid exposure to oxygen during liquid transfer or incubation. Optical density (OD) was measured in a 96-well plate at 200 μL per well by a plate reader (Synergy4, Biotek).

### RAW264.7 Macrophage Infection

One day prior to infection, RAW264.7 cells were seeded in 24-well plates at 1 mL per well with 2.5 × 10^5^ cells per well. For overnight propionate pretreatment of macrophages, propionate was added during seeding. For short propionate pretreatment of macrophages, propionate was added for 3 h prior to infections. None of the overnight or short pretreatments of propionate dramatically altered the macrophage confluency under microscopic inspections prior to the addition of bacteria for infection. The propionate concentrations used in this study did not cause any toxicity based on lactate dehydrogenase release assays using the commercially available LDH kits (BioVision K313500) following manufacturer’s protocols (data not shown). To prepare for the bacterial inoculum, *L. monocytogenes* cultures were harvested by centrifugations and washed with DPBS+/+ (VWR Life Sciences 02-0117-0500) to remove residual propionate. Infection inoculum was prepared with fresh DMEM and appropriate number of *L. monocytogenes* to achieve a multiplicity of infection (MOI) of 10 and added to the macrophages at 0.5 mL per well. After 30 min of infections, media in the well was aspirated and the cells were rinsed twice with DPBS+/+. Fresh media containing gentamicin (10 μg/mL; Gibco 15710-064) with or without propionate was added into the wells at 1 mL per well to eliminate extracellular *L. monocytogenes*.

### Intracellular CFU Calculation

Infection inoculum, consisting of DMEM and *L. monocytogenes*, was serially diluted and plated on LB agar plates to determine the input CFU per well. At 2 or 6 h post infection, infected macrophages were rinsed twice with DPBS+/+ and lysed with 200 μL of filter-sterilized Triton-X (0.1%, v/v) per well to release intracellular bacteria. The resulting lysates were serially diluted and plated on LB agar plates. CFUs were counted and used to calculate intracellular CFU per well. Percent input was calculated by comparing the intracellular CFU per well at 2 hpi and input CFU per well. Fold increase was calculated by comparing the intracellular CFU per well between 2 and 6 hpi.

### Actin Colocalization

One day prior to infection, autoclaved glass coverslips were placed into 6-well plates and seeded with 2 mL of RAW264.7 macrophages at 1 × 10^6^ cells per well. Short or overnight propionate treatments were performed as described earlier. Cells were infected at an MOI of 10 with 1 mL per well for 30 min and then rinsed twice with DPBS+/+. Fresh medium containing gentamicin (10 μg/mL) with or without propionate was added to the well at 2 mL per well. After 4 h of infection, cells were fixed in paraformaldehyde (4%, v/v, in PBS+/+) prior to staining. For immunofluorescence microscopy, coverslips containing the fixed cells were rinsed thoroughly with TBS-T (20 mM Tris–HCl, 150 mM NaCl, 0.1% [v/v] Triton X-100) and blocked with TBS-T with 1% (w/v) BSA for 30 min. Coverslips were then stained with primary *L. monocytogenes* antibody (Thermo Scientific PA1-30487) for 1 h, rinsed with TBS-T and treated with secondary antibodies (Abcam ab150077) and Phalloidin (Chem Cruz sc-363795) for 1 h. Coverslips were rinsed a final time with TBS-T and mounted to slides with ProLong Diamond Antifade Mountant with DAPI (Invitrogen P36966). Slides were allowed to set in the dark at room temperature overnight and then stored at 4°C until ready to view. Slides were viewed on a fluorescence microscope (Olympus BX51) at a 100× magnification. A minimum of 200 *L. monocytogenes* cells were counted for each replicate and condition. Percent co-localization was calculated as the number of *L. monocytogenes* with actin clouds divided by the total number of *L. monocytogenes* counted.

### Plaque Assay

L-fibroblast cells were harvested by trypsin (Gibco 25200-056) digest and resuspended in fresh media at 5 × 10^6^ cells per mL. The suspension was added to 6-well plates at 2 mL per well and incubated for 48 h to form a monolayer prior to infections. Overnight cultures (1 mL) of *L. monocytogenes* were normalized by culture OD, washed, and resuspended in 1 mL of DPBS+/+. Fresh DMEM media containing *L. monocytogenes* (6 μL of undiluted, 1:10, or 1:100 diluted suspension) were added to each well at 1 mL per well. At 1 hpi, media was aspirated off and a 3 mL overlay of DMEM with 0.7% (w/v) agarose and 10 μg/mL gentamicin was added into each well. At 3 days post infections (dpi), wells were stained with filter-sterilized neutral red (0.3%, w/v) in DMEM for 1 h. Wells were then rinsed with DPBS+/+ and left to develop overnight in the cell culture incubator. Plaques were measured at the widest point using Adobe Photoshop. A minimum of 80 plaques were counted for each condition over five independent experiments.

### Nitrite Assay

The level of nitrite is measured as an indicator for nitric oxide (NO) production. RAW264.7 cells, with or without activation by 1 ng/mL LPS (Sigma-Aldrich L4391) and 10 μg/mL IFN-γ (Fisher 50-253-689) in phenol-free DMEM (VWR 16777-406), were seeded in 24-well tissue culture plates at 1 mL per well and 2.5 × 10^5^ cells per well. Cells were treated with varying concentrations of propionate (0, 0.1, 1.0, or 10 mM) for 3 h or overnight incubation. NO production of these cells was determined by measuring the nitrite concentration in the cell culture media. In brief, 100 μL of cell culture supernatant was mixed with 100 μL of Griess reagent (1:1 of 1% [w/v] sulfanilamide in water and 0.1% [w/v] naphthyl ethylenediamine dihydrochloride in 10% [v/v] hydrochloric acid), which was made fresh on a weekly basis. Absorbance was measured after incubation at room temperature for 5 min using a 96-well plate reader (BioTek) at 560 nm. A potassium nitrite (KNO_2_) standard curve was used to calculate nitrite concentrations in the samples.

### Cell Shape Determination

RAW264.7 cells were seeded overnight in 24-well plates at 1 mL and 2.5 × 10^5^ cells per well with or without LPS (1 ng/mL) and IFNγ (10 μg/mL) for activation and for 3 h with 0, 1.0, or 10 mM propionate. Images were taken with an inverted microscope (Motic AE2000). Length and width of 10 cells per image were measured using ImageJ to calculate the length-to-width ratios.

### Migration Assay

Microfluidic devices (Figures 3B,C) were fabricated using previously established methods (Bui et al., 2018) and were taped to prevent entry of dust into the channels during storage. Prior to use, the devices and coverslips were sterilized in 70% (v/v) ethanol for 15 min, followed by 3 additional washes with sterile double-deionized water inside a biosafety cabinet (Nuaire LabGard ES Class II, Type A2). The sterilized devices were placed inside the biosafety cabinet to dry overnight. Sterile and dry devices were placed on coverslips to create intact channels. To remove air bubbles that could adversely affect cell viability and migration, the device reservoirs were filled with sterile DI water then vacuumed for 2–3 min using a desiccator. After removing all air bubbles, DI water was aspirated from the reservoirs prior to introducing cells. RAW264.7 cells with or without LPS (1 ng/mL) and IFNγ (10 μg/mL) for activation and propionate (0, 1.0, or 10 mM) were seeded into one reservoir of the devices at a concentration of 1.5 × 10^5^ cells per device. The remaining reservoir of each device was filled with DMEM with or without activation or propionate so that there was no chemical gradient. Microfluidic devices containing cells were incubated for 21 h and then fixed with 4% (v/v) paraformaldehyde for 15 min followed by three rinses with DPBS−/− (Corning 21-031-CV) and filled with DPBS−/− for storage. Images were taken with an inverted microscope and the number of cells within 200 μm of channel entry were counted using Adobe Photoshop.

### Statistical Analysis

Statistical analyses were performed in Microsoft Excel with *p-*values calculated by two-tailed Student’s *t-*tests and error bars representing standard errors of the mean. Outliers were identified and removed using the ESD method.

## Results

### Anaerobic Propionate Pretreatment of *L. monocytogenes* Enhanced Subsequent Intracellular Infections

**FIGURE 1:**
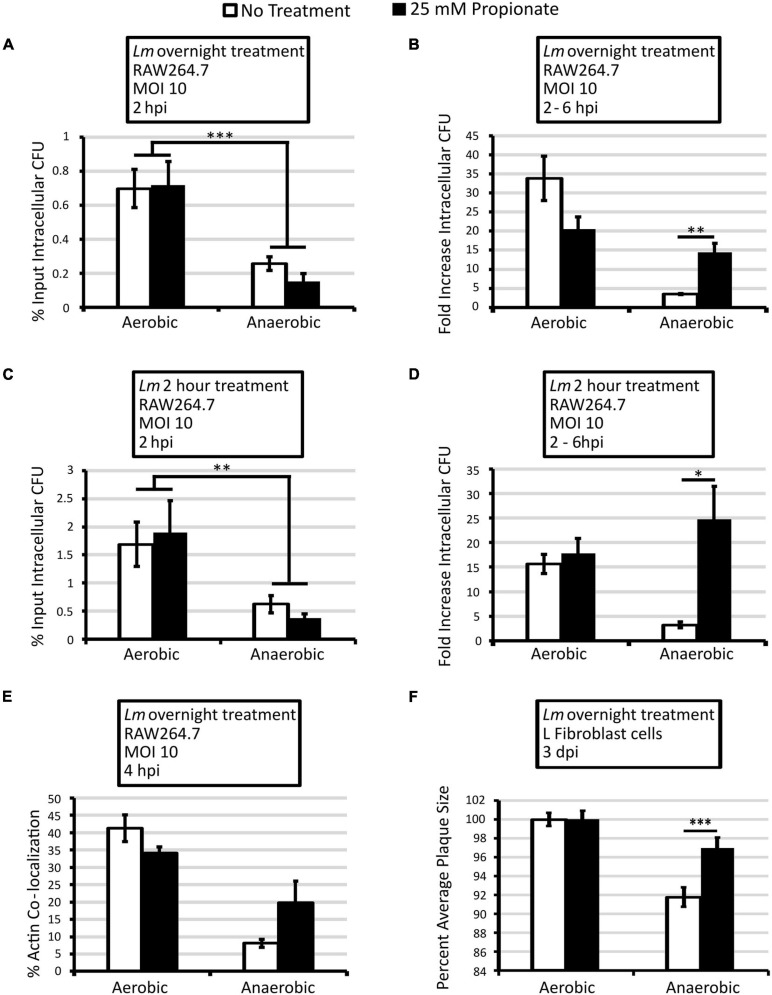
Pretreatment of anaerobic *L. monocytogenes* with 25 mM propionate enhances intracellular growth and cell-to-cell spread. *L. monocytogenes* strain 10403s was grown aerobically or anaerobically with (black bars) or without (white bars) 25 mM propionate for overnight **(A,B,E,F)** or for 2 h **(C,D)** and rinsed to remove all residual propionate from culture media prior to infections. Infected RAW64.7 macrophages were lysed at 2 and 6 hpi to enumerate intracellular CFU. Percent input intracellular CFU **(A,C)** was calculated by comparing intracellular CFU at 2 hpi to CFU in the infection inoculum while fold increase intracellular CFU **(B,D)** was calculated by comparing intracellular CFU between 2 and 6 hpi. Averages from 3 independent experiments, each performed in triplicate, were plotted with error bars representing standard errors of the mean **(A–D)**. **(E)** Immunofluorescence microscopy was performed at 4 hpi where filamentous actin and *L. monocytogenes* were differentially stained and quantified for co-localization. A minimum of 200 *L. monocytogenes* cells were counted from each replicate and condition over 3 independent experiments performed in duplicate. Averages were plotted with error bars representing standard errors of the mean. **(F)** Plaque diameters of infected L fibroblasts were measured at 3 dpi and those from aerobically grown *L. monocytogenes* without propionate were used as controls. Averages of a minimum of 80 plaques for each condition over 4 independent experiments were plotted with error bars representing standard errors of the mean. Asterisks denote statistical significance between propionate treated and control groups with ^∗^*p* < 0.05, ^∗∗^*p* < 0.01, and ^∗∗∗^*p* < 0.001.

To better understand the effects of propionate on *L. monocytogenes* infections, we first investigated how propionate exposures in *L. monocytogenes* prior to infection, such as those taking place in the food matrices or in the host intestinal lumen, affect subsequent intracellular growth using RAW264.7 macrophages and L-fibroblasts. RAW264.7 macrophages were infected by *L. monocytogenes* grown overnight with or without propionate and lysed at 2 and 6 hpi to enumerate intracellular CFU. Intracellular CFU at 2 hpi represented the degree of initial bacterial entry and survival and was normalized to the infection inoculum as percentages of input CFU to better compare across different infection conditions. Fold change in intracellular CFU between 2 and 6 hpi was then calculated to represent the degree of intracellular growth during primary infections. While an overall lower level of intracellular CFU was observed in macrophages infected by anaerobically grown *L. monocytogenes* compared to those infected by aerobically grown *L. monocytogenes*, overnight propionate pretreatment exhibited no significant effects on bacterial entry and initial survival (Figure 1A). However, overnight propionate pretreatment of anaerobically, not aerobically, grown *L. monocytogenes* significantly enhanced intracellular growth between 2 and 6 hpi (Figure 1B). For aerobically grown bacteria, propionate pretreatment resulted in a notable but not statistically significant decrease in intracellular growth across three independent experiments (Figure 1B). These results suggest that propionate pretreatment in *L. monocytogenes* does not affect the initial entry and survival in macrophages but can influence subsequent intracellular growth.

The lack of effects observed in aerobically grown bacteria led us to consider the possibility of propionate metabolism potentially depleting the exogenous propionate and diminishing the impact of overnight pretreatment. Therefore, effects of short-term propionate pretreatment were examined by back-diluting overnight, no-propionate cultures to fresh media for a 2-h propionate pretreatment of *L. monocytogenes* prior to infection. Similarly to overnight pretreatment, 2-h propionate pretreatment did not cause a significant difference in bacterial entry and initial survival at 2 hpi (Figure 1C) but resulted in a significant increase in intracellular growth between 2 and 6 hpi for anaerobically but not aerobically grown *L. monocytogenes* (Figure 1D). Compared to no propionate controls, overnight and 2-h propionate treatments enhanced intracellular growth of anaerobic *L. monocytogenes* approximately 4 and 8-fold, respectively. It is important to note that all bacteria were washed to remove any residual propionate prior to infection. Propionate was also omitted throughout the infection procedure. Therefore, the observed effects in propionate-pretreated bacteria are strongly indicative of a long-term impact on *L. monocytogenes* from anaerobic propionate exposure beyond initial interactions with the host cells.

To identify potential contributing factors in the enhanced intracellular growth by anaerobic, propionate-pretreated bacteria, actin co-localization, a cytosolic process during *L. monocytogenes* intracellular life cycle, was measured at 4 hpi to determine the effects of propionate pretreatment on phagosomal escape. When overnight *L. monocytogenes* were used to infect RAW264.7 macrophages, no significant difference was observed in percentages of actin co-localization between propionate-pretreated bacteria and no treatment controls (Figure 1E). For anaerobically grown *L. monocytogenes*, a notable increase was observed for propionate-treated bacteria, but the difference was not statistically significant when averaging across 3 independent experiments (Figure 1E). Therefore, the distinctive impact of propionate on LLO production *in vitro* (Rinehart et al., 2018) was not sufficient to cause a significant change in phagosomal escape. Moreover, the enhanced intracellular growth exhibited by anaerobic, propionate-treated bacteria (Figure 1B) was likely not attributed to increased entry into the replicative niche.

To establish the impact of prior propionate exposure beyond primary infections, plaque assays were performed where monolayers of murine L-fibroblasts were infected by overnight *L. monocytogenes* and plaque diameters were measured at 3 dpi as indicators of intracellular growth and cell-to-cell spread. Similar to macrophage infections, bacteria were washed to remove residual propionate so that propionate exposure took place only during bacterial overnight growth but not fibroblast infections. At 3 dpi, propionate pretreatment did not affect plaque sizes for aerobically grown *L. monocytogenes* but resulted in significantly larger plaques for anaerobically grown *L. monocytogenes* (Figure 1F), thereby alleviating the infection defect of anaerobically grown *L. monocytogenes*. Altogether, these results indicate that exposure of *L. monocytogenes* to propionate under anaerobic, but not aerobic, conditions for as short as 2 h prior to infection causes significantly enhanced intracellular infections particularly after bacterial entry into the host cells.

### Anaerobic Propionate Pretreatment of *L. monocytogenes* Enhanced NO Production in Infected Macrophages

**FIGURE 2:**
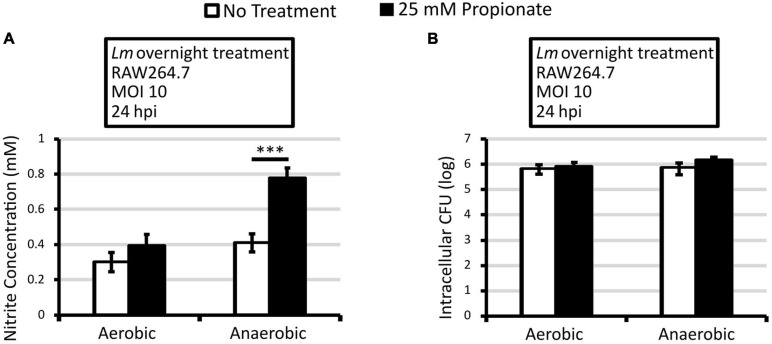
Macrophages infected with *L. monocytogenes* grown anaerobically with propionate pretreatment produce higher levels of NO. Overnight cultures of *L. monocytogenes* were washed and used to infect RAW264.7 macrophages for 30 min. No propionate was present during infections. Nitrite concentration **(A)** and intracellular CFU **(B)** were measured at 24 hpi. Averages of 9 replicates across 3 independent experiments were plotted with error bars representing standard errors of the mean. Asterisks denote statistical significance between pair-wise comparisons with ^∗∗∗^*p* < 0.001.

It has been established previously that NO enhances secondary *L. monocytogenes* infection and actin polymerization (Cole et al., 2012; McFarland et al., 2018). Therefore, to determine whether the enhanced intracellular infections by anaerobic, propionate-pretreated *L. monocytogenes* was a result of increased NO production by macrophages, nitrite concentration was quantified from the supernatant of infected macrophages at 24 hpi. Significantly higher levels of nitrite were observed in macrophages infected with anaerobic, propionate-pretreated *L. monocytogenes* compared to macrophages infected with anaerobic bacteria without propionate treatments (Figure 2A). This was not a result of higher bacterial burden because similar levels of intracellular CFU were observed at 24 hpi (Figure 2B). Therefore, in addition to enhancing infections by anaerobically grown bacteria, propionate pretreatment in *L. monocytogenes* also resulted in increased macrophage NO production. Again, it is important to note that no propionate was present during the infections. Therefore, the significant increase in nitrite levels (Figure 2A) further suggests the long-lasting impact of prior anaerobic propionate exposure on subsequent interactions between *L. monocytogenes* and the host cells. Moreover, these results also suggest that macrophages likely have the capability to distinguish and respond differently to *L. monocytogenes* with prior anaerobic propionate exposure.

### Propionate Treatment Altered Cell Morphology and Reduced NO Production in Uninfected Macrophages

**FIGURE 3:**
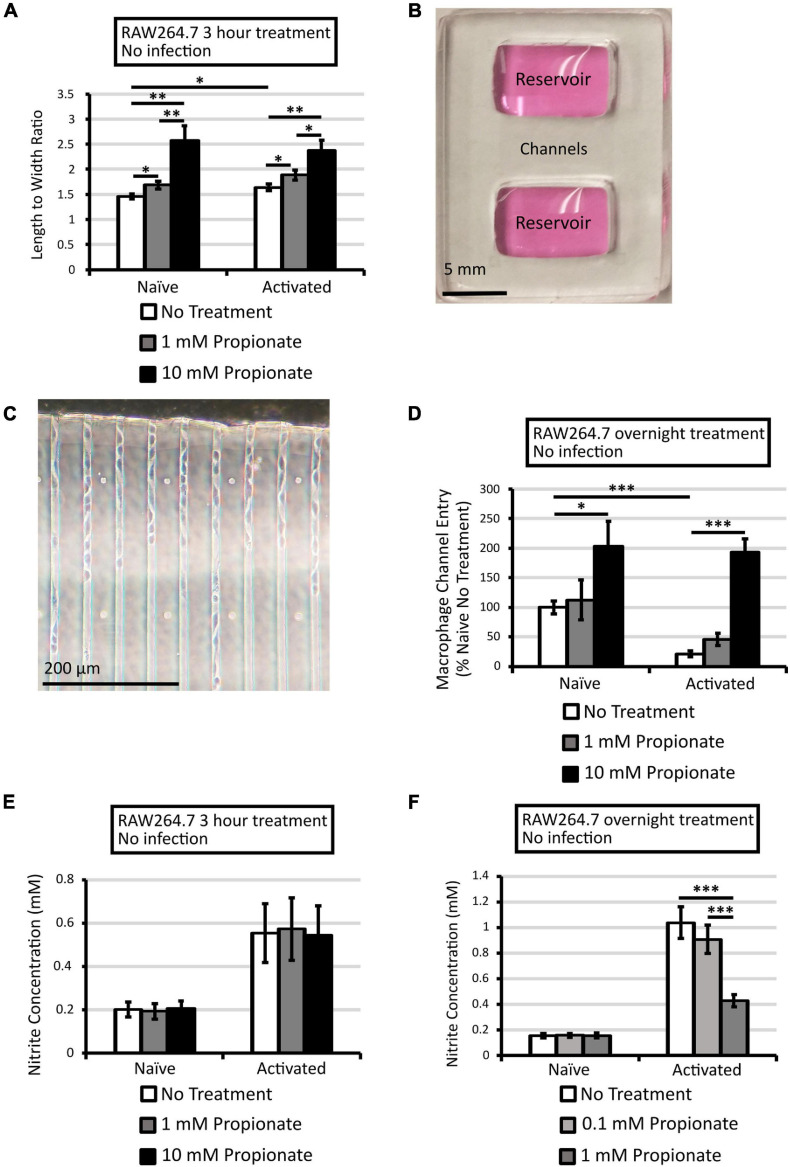
Propionate treatment alters RAW264.7 activation status. To assess macrophage activation status, morphology and nitrite concentrations were measured for macrophages with or without activation by LPS and IFNγ. To assess morphology, microscopic images of macrophages with or without propionate treatment for 3 h were analyzed with ImageJ where cell length-to-width ratios of at least 20 cells across 2–4 independent experiments were calculated with error bars representing standard errors of the mean **(A)**. Additionally, entry into microfluidic channels was assessed using microfluidic devices that contain two reservoirs connected by channels with a cross section of 10 × 10 μm **(B,C)**. RAW264.7 macrophages were seeded into a reservoir with or without activation and 0, 1, or 10 mM propionate and fixed after 21 h for image analysis. Averages of cell counts in channels up to 200 μm from the entry point from two independent experiments (108 channels in total) were plotted with error bars representing standard errors of the mean **(D)**. Nitrite concentration was quantified for RAW264.7 macrophages treated with propionate for 3 h **(E)** or overnight **(F)**. Averages of 3 **(E)** and 4 **(F)** independent experiments, each performed in triplicate, were plotted with error bars representing standard errors of the mean. Asterisks denote statistical significance between pair-wise comparisons with ^∗^*p* < 0.05, ^∗∗^*p* < 0.01, and ^∗∗∗^*p* < 0.001.

To better understand how propionate might influence macrophage activities against *L. monocytogenes*, we first compared the effects of propionate on naïve and activated macrophages in the absence of infections. Macrophages were activated overnight with LPS and IFNγ and then treated with propionate for 3 h. Microscope images of these cells were analyzed by ImageJ where the cell length-to-width ratio was calculated. Propionate treatment, in a dose-dependent manner, resulted in significantly higher length-to-width ratios in both naïve and activated macrophages (Figure 3A). These morphological alterations were also reflected by the enhanced entry into microfluidic channels (Figures 3B–D). Without propionate treatment, naïve macrophages exhibited higher levels of entry into channels than activated macrophages (Figure 3D). For both the naïve and activated macrophages, propionate treatment at 10 mM significantly increased in the number of macrophages entering the channels (Figure 3D). While the elongating effects of propionate on cell shape were observed independently of macrophage activation status, the effects of propionate on NO production were only observed in activated macrophages (Figures 3E,F). For naïve macrophages, the presence of propionate did not cause any significant changes in culture nitrite levels. In contrast, while 3 h of propionate treatment up to 10 mM was not sufficient to cause a change in culture nitrite levels (Figure 3E), overnight incubation with 1 mM propionate was sufficient to cause significantly lower nitrite levels compared to no propionate controls in activated macrophages (Figure 3F). These results align with the known anti-inflammatory role of propionate in suppressing macrophage activation, such as the reduced NO production, that can potentially alter subsequent susceptibility to intracellular *L. monocytogenes* infections.

### Short Propionate Pretreatment Did Not Alter NO Production, but Lowered Infection by Anaerobic *L. monocytogenes*

**FIGURE 4:**
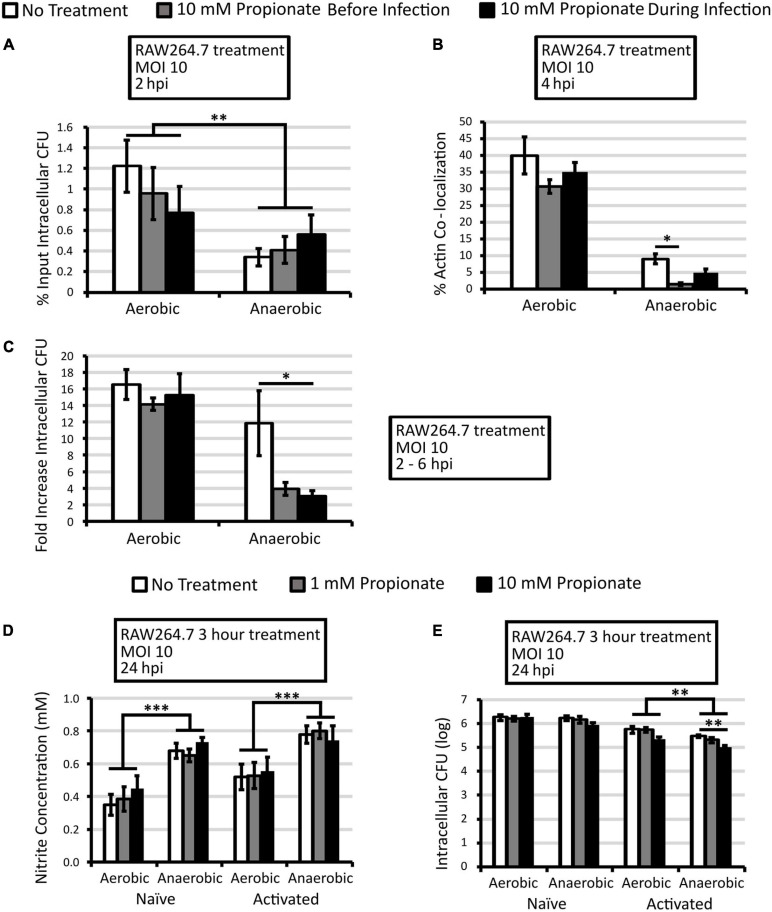
Short propionate pretreatment did not alter NO production by infected macrophages but reduces anaerobic *L. monocytogenes* infection. **(A–C)** RAW264.7 macrophages were incubated with or without 10 mM of propionate for 3 h prior to infection (gray bars) or during infection (black bars) by aerobically or anaerobically grown *L. monocytogenes.* Infected macrophages were lysed at 2 and 6 hpi to enumerate intracellular CFU. Percent input intracellular CFU **(A)** was calculated by comparing intracellular CFU at 2 hpi to CFU in the infection inoculum. Immunofluorescence microscopy was performed at 4 hpi **(B)** where filamentous actin and *L. monocytogenes* were differentially stained and quantified for co-localization. A minimum of 200 *L. monocytogenes* cells were counted from each replicate and condition over 2 independent experiments performed in duplicate. Averages were plotted with error bars representing standard errors of the mean **(B)**. Fold increase intracellular CFU **(C)** was calculated by comparing intracellular CFU between 2 and 6 hpi. Averages from 4 independent experiments, each performed with triplicate, were plotted with error bars representing standard errors of the mean **(A,C)**. Nitrite concentrations in culture supernatant were quantified for RAW264.7 macrophages treated with or without activation by LPS and IFNγ overnight and with propionate for 3 h prior to infections. Overnight aerobically or anaerobically grown *L. monocytogenes* were used to infect macrophages for 30 min where supernatant nitrite levels **(D)** and intracellular CFUs **(E)** were measured at 24 hpi. Averages are from at least 3 independent experiments, each performed in triplicate, with error bars representing standard errors of the mean **(D,E)**. Asterisks denote statistical significance between pair-wise comparisons with ^∗^*p* < 0.05, ^∗∗^*p* < 0.01, and ^∗∗∗^*p* < 0.001.

The observed role of propionate in macrophage morphology and NO production (Figure 3) prompted us to investigate the impact of propionate on the ability of macrophages to control *L. monocytogenes* intracellular growth. Therefore, macrophages were treated with 10 mM propionate either for 3 h prior to or during infections. Intracellular CFUs were then quantified at 2 and 6 hpi. At 2 hpi, there was no significant impact of propionate treatment in macrophages on initial entry and survival of aerobic or anaerobic *L. monocytogenes* (Figure 4A). However, there is a significantly higher level of entry and survival of aerobic than anaerobic *L. monocytogenes* (Figure 4A). At 4 hpi, actin polymerization by *L. monocytogenes* was significantly reduced in pretreated macrophages infected with anaerobic *L. monocytogenes* compared to untreated macrophages (Figure 4B). Furthermore, between 2 and 6 hpi, treatment of macrophages, either before or during infections, significantly inhibited intracellular growth of anaerobic but not aerobic *L. monocytogenes* (Figure 4C). These results suggest that the presence of propionate outside of host cells can potentially influence *L. monocytogenes* intracellular infections by acting directly on intracellular bacteria or through modulating macrophage antimicrobial functions.

To determine if a 3-h propionate pretreatment of macrophages was sufficient to cause sustained effects on infection outcomes, naïve and activated macrophages were treated for 3 h with propionate and then infected by overnight *L. monocytogenes*. Propionate was removed from the cell culture media prior to infection. At 24 hpi, no significant differences were observed in nitrite levels between no propionate control and propionate treatment groups (Figure 4D). However, significantly higher nitrite levels were observed consistently in macrophages infected by anaerobically grown *L. monocytogenes* compared to those infected by aerobically grown *L. monocytogenes* (Figure 4D). No significant differences in intracellular CFUs were observed in infected naïve macrophages with or without propionate pretreatment (Figure 4E). In activated macrophages, in contrast, propionate pretreatment at 10 mM resulted in a significant decrease in intracellular CFUs for infections by anaerobically grown *L. monocytogenes*. Together, these results suggest an opposing effect of propionate on *L. monocytogenes* and macrophages so that while propionate exposure of anaerobic *L. monocytogenes* results in enhanced infections, propionate exposure of macrophages, before or during infections, results in compromised infections by anaerobic *L. monocytogenes*.

### Propionate Treatment During Infection Was Protective Against Propionate-Treated *L. monocytogenes*

**FIGURE 5:**
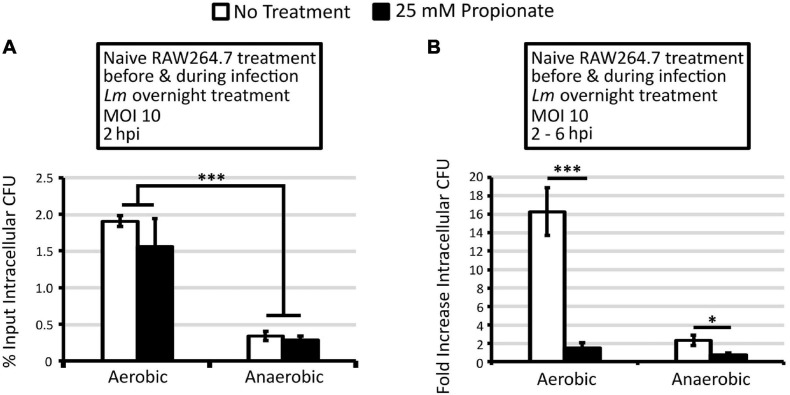
Treatment of macrophages and aerobic or anaerobic *L. monocytogenes* with 25 mM propionate reduces intracellular growth. *L. monocytogenes* strain 10403s was grown aerobically or anaerobically with or without 25 mM propionate overnight and rinsed to remove all residual propionate from culture media prior to infections. RAW264.7 macrophages were incubated with or without 25 mM of propionate for 3 h prior to infection and during infections. White bars represent no treatment and black bars represent combined propionate treatment of both *L. monocytogenes* and macrophages. Infected macrophages were lysed at 2 and 6 hpi to enumerate intracellular CFU. Percent input intracellular CFU **(A)** was calculated by comparing intracellular CFU at 2 hpi to CFU in the infection inoculum. Fold increase intracellular CFU **(B)** was calculated by comparing intracellular CFU between 2 and 6 hpi. Averages from 3 independent experiments, each performed in triplicates, were plotted with error bars representing standard errors of the mean **(A,B)**. Asterisks denote statistical significance between pair-wise comparisons with ^∗^*p* < 0.05 and ^∗∗∗^*p* < 0.001.

The opposing effect of propionate treatment of RAW264.7 cells and anaerobic *L. monocytogenes* on intracellular growth led us to investigate the impact of treating both macrophages and *L. monocytogenes* on the infection outcomes. RAW264.7 macrophages treated with or without propionate (25 mM) for 3 h were infected with *L. monocytogenes* grown overnight with or without propionate (25 mM). During infection, propionate (25 mM) was also added to the medium along with gentamicin so that propionate-treated *L. monocytogenes* would continue to be exposed to propionate added to the cell culture medium during infections while bacteria and macrophages in no treatment controls were not exposed to any propionate throughout the experiment. Intracellular CFUs were enumerated at 2 and 6 hpi to determine *L. monocytogenes* entry and survival early in the infection as well as the capacity for intracellular growth. At 2 hpi, similarly to earlier results (Figures 1A, 4A), simultaneous propionate treatments of macrophages and *L. monocytogenes* did not significantly alter initial entry and survival (Figure 5A). As seen previously, aerobic *L. monocytogenes* exhibited a significantly higher level of intracellular CFU at 2 hpi than anaerobic *L. monocytogenes* (Figure 5A). Between 2 and 6 hpi, simultaneous propionate treatments of macrophages and *L. monocytogenes* significantly inhibited intracellular growth for both aerobic and anaerobic *L. monocytogenes* (Figure 5B). These results suggest that while separate propionate treatments did not result a significant impact on intracellular growth of aerobic *L. monocytogenes*, the combined effects of propionate on macrophages and aerobic *L. monocytogenes* are sufficient to limit intracellular growth. Moreover, the protective effects of propionate treatment of macrophages are sufficient to negate the enhancing effects of propionate treatment of anaerobic *L. monocytogenes*.

## Discussion

In this study, we investigated the effects of propionate on the interactions between *L. monocytogenes* and two different host cell models and found that propionate exposure, whether taking place before or during infections, could significantly impact infection outcomes. For *L. monocytogenes*, anaerobic propionate exposure significantly enhanced subsequent intracellular growth. Independently from infections, RAW264.7 macrophages also responded readily to propionate by altering NO production and cell morphology. During macrophage infections, the presence of propionate significantly compromised *L. monocytogenes* intracellular growth. Together, these results highlighted the potential for propionate as an important modulator of *L. monocytogenes* pathogenesis and host responses. These findings support the need to investigate additional host infection models, such as primary macrophages, intestinal epithelial cell lines, or animals, to obtain a more comprehensive understanding of the role of propionate in *L. monocytogenes* infections.

### Propionate Exposure at Different Stages of Infection

Results from this study highlighted the importance of propionate exposure throughout the infection process. Prior to infection, *L. monocytogenes* is potentially exposed to propionate in food at concentrations ranging from 3,000 mg/kg (in baked goods and cheese) to 5,000 mg/kg (in processed meats and fish; Kagliwal et al., 2014; European Food Safety Authority (EFSA), 2016). Similarly, during intestinal transit, *L. monocytogenes* can be exposed to 10–100 mM of SCFAs in the lumen (Natarajan and Pluznick, 2014; Koh et al., 2016). The infected host cells are also exposed to SCFAs at concentrations between 0.1 and 10 mM (Natarajan and Pluznick, 2014; Koh et al., 2016). Therefore, using physiologically relevant levels of propionate, we identified various processes during host-pathogen interactions that were significantly impacted by propionate. First, anaerobic but not aerobic propionate exposure in *L. monocytogenes* prior to infection resulted in a significantly enhanced intracellular infection beyond the initial interactions (Figure 1). This observation highlighted the importance of anaerobic adaptation to propionate by *L. monocytogenes*, which might take place during food storage and intestinal transit, in establishing subsequent intracellular life cycle. In contrast, propionate exposure during infections significantly compromised infections, particularly by propionate-pretreated *L. monocytogenes*, a result indicative of macrophage responses to propionate separately from virulence regulations in pathogens and arguing for additional studies in other host models to gain a better understanding of the regulatory functions of propionate.

RAW264.7 macrophages are a commonly used cell line to investigate *L. monocytogenes* intracellular infections, they do not represent the full range of cell types *L. monocytogenes* encounters to establish infections in a host. Most notably, given the high propionate levels inside the intestinal lumen, the role of propionate in *L. monocytogenes* invasion of the intestinal epithelium remains to be determined. While propionate exposure strongly regulates LLO production (Rinehart et al., 2018), whether it similarly regulates other virulence factors necessary for *L. monocytogenes* to survive the mucosal defenses and cross the epithelial layer to establish infections in peripheral organs is unknown. Several lines of evidence supported the role of propionate supplementations in directly influencing phenotypes of colonic epithelial cells (Wilson and Gibson, 1997; Malago et al., 2003; Fu et al., 2004; Kilner et al., 2012), including a decrease in paracellular permeability (Mariadason et al., 1997; Suzuki et al., 2008; Feng et al., 2018) and the production of proinflammatory cytokines (Hung and Suzuki, 2018). These modifications can potentially alter cellular and host susceptibility to *L. monocytogenes* infections but need to be experimentally determined.

### Propionate Metabolism by Anaerobically Grown *L. monocytogenes*

Propionate is a three-carbon carboxylic acid that can be readily metabolized by *L. monocytogenes* for membrane fatty acid synthesis (Rinehart et al., 2018). Under anaerobic conditions, we have reported that *L. monocytogenes* exhibited a distinctively different surface morphology under transmission electron microscopy compared to those grown under aerobic conditions (Wallace et al., 2017). Although the proportions of branch chain fatty acids (BCFAs) generally decrease with propionate supplementation under both aerobic and anaerobic conditions, anaerobic but not aerobic propionate treatments significantly increased the anteiso- to iso-BCFA ratios (Rinehart et al., 2018). BCFAs as a whole play a role in *L. monocytogenes* resistance to phagosomal killing and other antimicrobial defenses (Sun et al., 2012). Anteiso BCFAs, in particular, are essential for intracellular survival and growth, but not initial uptake by macrophages (Sun and O’Riordan, 2010). Therefore, it is likely that propionate differentially affects anaerobic *L. monocytogenes* membrane composition by restoring the anteiso- to iso-BCFA ratios, an adaptation that persists after entry into a host cell cytosol to promote subsequent intracellular success even when propionate is absent.

**FIGURE 6:**
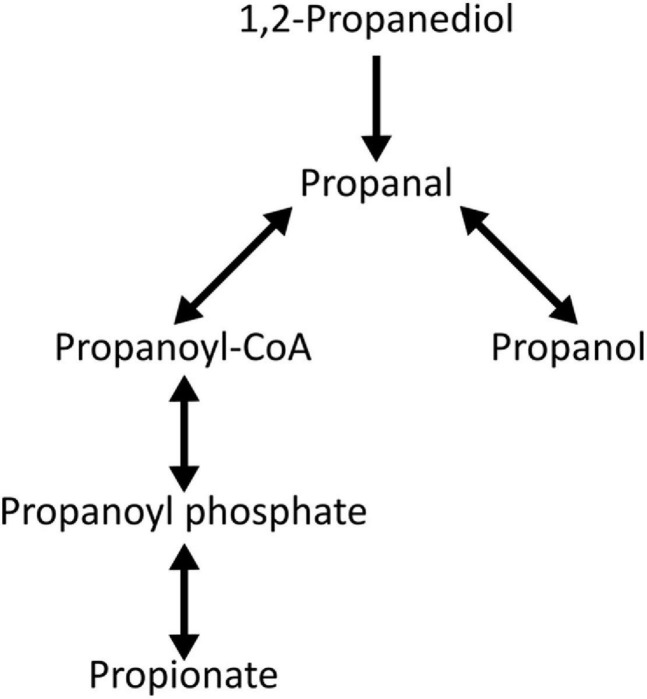
The 1,2-Propanediol degradation pathway in *L. monocytogenes* strain 10403s based on BioCyc Database Collection (BioCyc, 2012). Reversible pathway is indicated by double arrows.

Moreover, *L. monocytogenes* is capable of propionate production through the propanediol pathway (Figure 6; Koh et al., 2016; Zeng et al., 2019). More specifically, anaerobic *L. monocytogenes* can utilize the propanediol pathway to convert 1,2-propanediol into propionate and 1-propanol, using a cobalamin-dependent bacterial microcompartment (Koh et al., 2016; Zeng et al., 2019). Previous studies have shown that the ability of *L. monocytogenes* to utilize propanediol could enhance anaerobic growth *in vitro* (Zeng et al., 2019) and bacterial persistence *in vivo* (Schardt et al., 2017). Therefore, together with our observations, it is possible that propionate production, either through environmental sources or through 1,2-propanediol metabolism, is a critical intermediate to promote *L. monocytogenes* growth and survival inside the anaerobic lumen and to help *L. monocytogenes* transition into the intracellular niche.

### Propionate Responses in Macrophages

In the absence of *L. monocytogenes* infection, both naïve and LPS/IFNγ-activated RAW264.7 macrophages readily respond to propionate treatments by altering cell shape with increasing length-to-width ratios and entry into microfluidic channels (Figures 3A,D). These phenotypes are notably similar to observations in M2 macrophages, which typically exhibit an elongated cell shape and higher motility than naïve or M1 macrophages (McWhorter et al., 2013; Vogel et al., 2014). Naïve macrophages can be activated either classically into a proinflammatory M1 phenotype by IFNγ, TNF-α, and TLR ligands to defend against infection or into an anti-inflammatory M2 phenotype by IL-4 and IL-13 to aid in healing (Tan et al., 2016; Srivastava et al., 2017). There is a curious connection between macrophage activation and cell shape. For example, murine bone marrow derived macrophages (BMDMs) induced by IL-4 and IL-13 exhibited an elongated cell shape (McWhorter et al., 2013). Interestingly, forcing elongation through micropatterning can enhance arginase-1 and reduce iNOS expression, thereby stimulating cells toward an M2 phenotype in the absence of soluble cues (McWhorter et al., 2013). M2 macrophages from primary human monocyte-derived macrophages exhibit enhanced motility by traveling longer distances, better migration through dense 3D matrices, and higher random motility on polyacrylamide gel (Cougoule et al., 2012; Vogel et al., 2014; Hind et al., 2016). Therefore, propionate treatment in uninfected macrophages appears to mimic M2 phenotypes.

Propionate treatment results in a significantly reduced NO production in LPS and IFNγ-activated, uninfected macrophages (Figure 3F), an observation in alignment with the M2 phenotype and in support of the anti-inflammatory role of propionate. Similar to our observations in uninfected cells, propionate was shown to inhibit NO production in RAW264.7 cells stimulated with LPS or *S. aureus* lipoprotein (Liu et al., 2012; Park et al., 2019). In contrast, in infected macrophages the presence of anaerobically grown, *L. monocytogenes* generally induced a higher NO production in macrophages regardless of propionate treatment (Figure 4D). NO is known to benefit *L. monocytogenes* in certain infection situations. In BMDMs, NO, stimulated through TLR agonists LPS, Pam3CSK, PIC, and CpG^[+]^, inhibits primary infection but enhances secondary infections by delaying phagolysosome maturation in secondary infected cells (Cole et al., 2012). Similarly in RECON-deficient hepatocytes, NO production conveys an advantage to *L. monocytogenes* actin polymerization as seen by longer actin tails with a longer association period and rate of movement (McFarland et al., 2018). Interestingly, ActA expression is unaffected by the increased NO production, a result indicating the higher actin polymerization is due to host processes being impacted by higher NO levels (McFarland et al., 2018). In this study, it is likely that higher NO levels contributed to the enhanced infections by *L. monocytogenes* with prior anaerobic propionate treatments (Figure 2A). However, the significant decrease in intracellular CFU observed in activated macrophages (Figure 4E) despite the lack of effects on NO production after short term propionate pretreatment (Figure 4D) suggest that additional host factors, such as macrophage membrane lipid biosynthesis and composition that can be impacted by propionate (Costa Rosa et al., 1995), remain to be identified and are under investigation.

In conclusion, this study highlights the opposing role of propionate exposure on *L. monocytogenes* and macrophages in infection outcomes. Anaerobic exposures to propionate in *L. monocytogenes*, such as those taking place in food storage or intestinal transit in a host, could potentially enhance *L. monocytogenes* long-term survival and growth inside host cells. However, propionate exposure in macrophages that can occur in tissues or circulation could enhance the ability of macrophages to restrict the propagation of *L. monocytogenes*. Together, our findings establish propionate as an important modulator of *L. monocytogenes*-host interactions and highlight the need to include other host infection models and consider the conditions of exposure, such as the presence or absence of oxygen, for a more comprehensive understanding of propionate and its effects on foodborne infections.

## Data Availability Statement

The raw data supporting the conclusions of this article will be made available by the authors, without undue reservation.

## Author Contributions

YS, LH, and LB designed the research. LH, LA, MB, SJ, CD, and LB performed the experiments. YS and LH wrote the manuscript. All authors analyzed and interpreted data, reviewed the results, and approved the final version of the manuscript.

## Conflict of Interest

The authors declare that the research was conducted in the absence of any commercial or financial relationships that could be construed as a potential conflict of interest.

## Publisher’s Note

All claims expressed in this article are solely those of the authors and do not necessarily represent those of their affiliated organizations, or those of the publisher, the editors and the reviewers. Any product that may be evaluated in this article, or claim that may be made by its manufacturer, is not guaranteed or endorsed by the publisher.
